# P-1187. Olorofim for the treatment of Central Nervous System (CNS) invasive fungal infections (IFI) in patients with limited or no treatment options: a sub-analysis of an open-label, single-arm, Phase 2b trial (Study 32; NCT03583164)

**DOI:** 10.1093/ofid/ofaf695.1380

**Published:** 2026-01-11

**Authors:** Fariba Donovan, George R Thompson, Royce Johnson, Martin Hoenigl, Johan A Maertens, Andrej Spec, Andrea Deschambeault, Valerie Ravenna, Daniela Zinzi, Omar Fernandez, John H Rex

**Affiliations:** University of Arizona College of Medicine-Tucson, Tucson, Arizona; University of California, Davis Medical Center, Sacramento, California; Kern Medical Center, Bakersfield, California; Medical University of Graz, Graz, Austria, Graz, Steiermark, Austria; UZ Leuven, Leuven, Vlaams-Brabant, Belgium; Washington University School of Medicine in St. Louis, St. Louis, Missouri; F2G, Princeton, New Jersey; F2G, Princeton, New Jersey; F2G, Princeton, New Jersey; F2G, Princeton, New Jersey; F2G, Princeton, New Jersey

## Abstract

**Background:**

CNS IFIs are associated with high mortality rates. They affect immunocompetent and immunosuppressed hosts and are challenging due to limitations of licensed agents (poor CNS penetration; resistance).

**Table 1.**
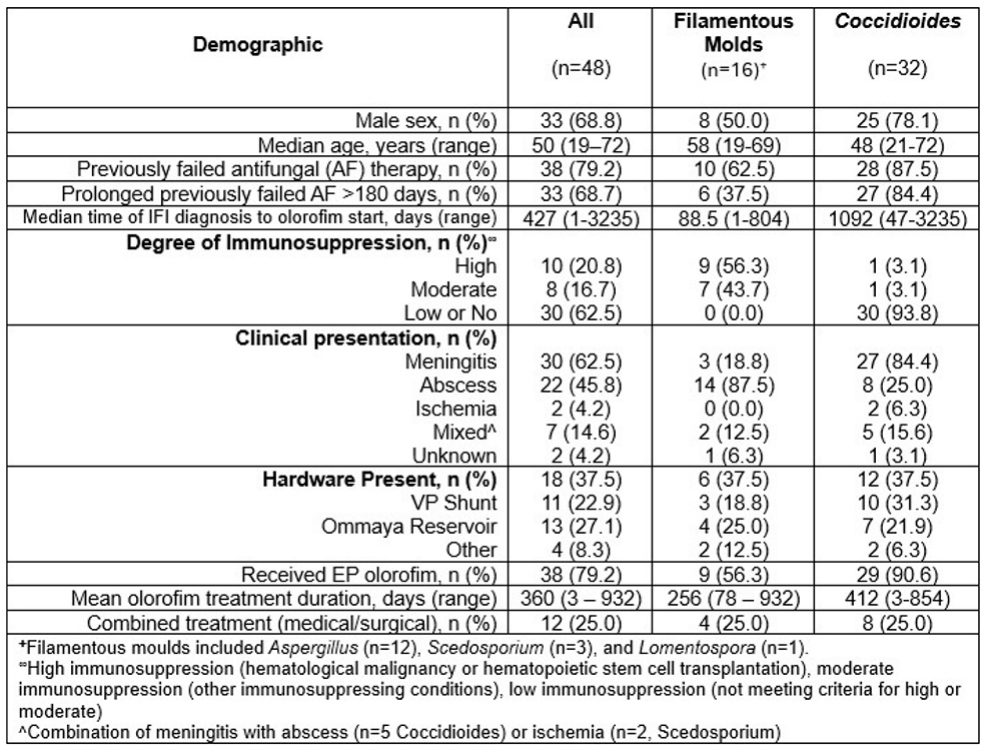
Baseline Characteristics of patients treated with olorofim in patients with CNS infection

**Table 2.**
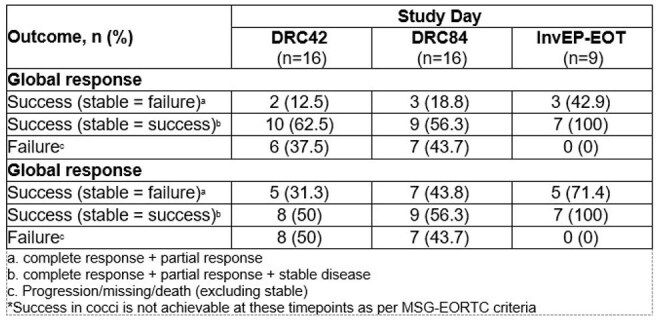
Outcomes in patients with CNS IFI, excluding coccidioidomycosis*

**Methods:**

Study 32, an open-label trial of oral olorofim (first-in-class orotomide with activity against difficult-to-treat rare and dimorphic moulds), included 48/203 patients with proven CNS IFI. Global response (GR; composite of radiological, mycological, and clinical responses [CR]) was adjudicated by a Data Review Committee (DRC; Day 42 and Day 84) or the investigator (> Day 90) using MSG/EORTC 2008 criteria.^a,b,c^ Olorofim beyond D90 was permitted in an Extended treatment Phase (EP).

**Table 3.**
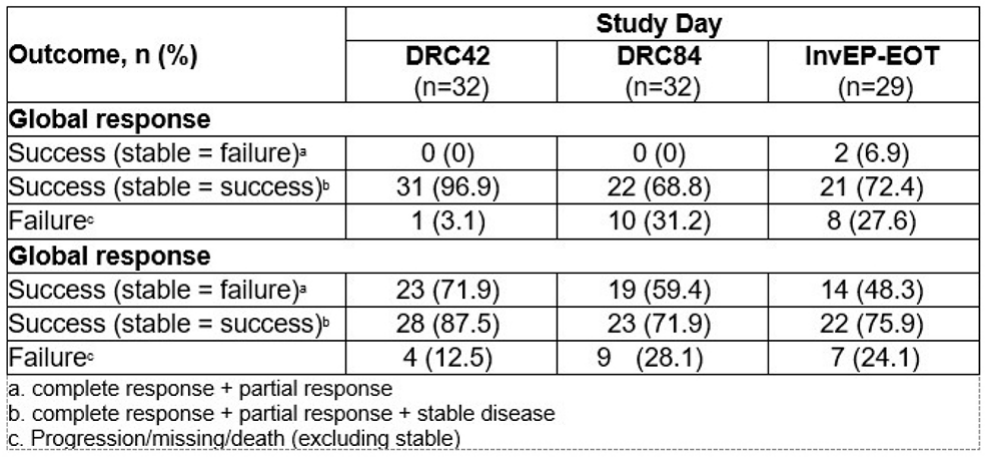
Outcomes in patients with coccidioidomycosis

**Results:**

Causative pathogens (Table 1) included *Coccidioides* (n=32), *Aspergillus* (n=12), *Scedosporium* (n=3), and *Lomentospora* (n=1). In patients with filamentous molds (FM; table 2), DRC-adjudicated successful GR was 12.5% (2/16) and 18.8% (3/16) at D42 and D84, respectively, when improvement was required in all three components. When stable disease was judged as success, DRC-adjudicated GR was 62.5% (10/16) and 56.3% (9/16) at D42 and D84, respectively. Successful CR, including stable disease, were seen in 50% (8/16) and 56.3% (9/16) at D42 and D84, respectively. In patients with coccidioidomycosis (table 3), GR was 0% at D42 and D84 due to slow resolution of serologic findings, even in those who achieved complete CR. For the 38 patients (*Coccidioides* n=29, FM n=9) who entered EP, Investigator-adjudicated CR at End of Treatment (InvEP-EOT) showed higher success and less failure rates than DRC D84, especially in patients with FM. All-cause mortality at both D42 and D84 was 31.3% (5/48); all deaths were in patients with FM IFI.

For the full cohort (N=203), the main AE was LFT alterations, judged at least possibly related to olorofim in 9.9% (20/203); 3% (6/203) requiring discontinuation.

**Conclusion:**

Oral olorofim was well tolerated with positive treatment outcomes in patients with CNS infections, even those on extended therapy. MSG/EORTC assessments at D42 and D84 may be insufficient to evaluate clinical outcomes, especially in patients infected with coccidioidomycosis.

**Disclosures:**

George R. Thompson III, MD, Astellas: Advisor/Consultant|Astellas: Grant/Research Support|Basilea: Advisor/Consultant|Basilea: Grant/Research Support|Cidara: Advisor/Consultant|Cidara: Grant/Research Support|F2G: Advisor/Consultant|F2G: Grant/Research Support|GSK: Advisor/Consultant|GSK: Grant/Research Support|Melinta: Advisor/Consultant|Melinta: Grant/Research Support|Mundipharma: Advisor/Consultant|Mundipharma: Grant/Research Support|Scynexis: Advisor/Consultant|Scynexis: Grant/Research Support Royce Johnson, MD, CDC: Travel|F2G: Advisor/Consultant|NIH: Grant/Research Support Martin Hoenigl, MD, AiCuris: Advisor/Consultant|F2G: Grant/Research Support|F2G: Honoraria|Gilead: Honoraria|Melinta: Grant/Research Support|Mundipharma: Advisor/Consultant|Mundipharma: Honoraria|Scynexis: Grant/Research Support|Shionogi: Honoraria Johan A. Maertens, MD PhD, Amgen: Consulting fees and non-financial support|Astellas Pharma: Honoraria|Astellas Pharma: Consulting fees and non-financial support|Basilea: Consulting fees and non-financial support|Bio-Rad: Grant/Research Support|Bio-Rad: Consulting fees and non-financial support|Cidara: Consulting fees and non-financial support|F2G: Honoraria|F2G: Consulting fees and non-financial support|Gilead Sciences: Grant/Research Support|Gilead Sciences: Honoraria|Gilead Sciences: Consulting fees and non-financial support|Merck: Grant/Research Support|Merck: Consulting fees and non-financial support|Merck Sharpe & Dohme: Honoraria|Mundipharma: Honoraria|Pfizer: Grant/Research Support|Pfizer: Honoraria|Pfizer: Consulting fees and non-financial support|Schering-Plough: Consulting fees and non-financial support|Scynexis: Consulting fees and non-financial support|Shire/Takeda: Consulting fees Andrej Spec, M.D., MSCI, Astellas Global Development Pharma, Inc: Grant/Research Support|Cidara: Grant/Research Support|Mayne Pharma: Grant/Research Support|Scynexis: Grant/Research Support Andrea Deschambeault, PharmD, F2G: Employee of company Valerie Ravenna, PharmD, F2G: Employee of company Daniela Zinzi, MD, F2G: Employee of company Omar Fernandez, MD, F2G: Employee of company John H. Rex, MD, F2G Limited: Employee|F2G Limited: Stocks/Bonds (Private Company)

